# Editorial: Effects of tobacco use on oral health

**DOI:** 10.3389/froh.2026.1803555

**Published:** 2026-03-06

**Authors:** Praveen Jodalli, Mamata Hebbal

**Affiliations:** 1 Department of Public Health Dentistry, Manipal College of Dental Sciences Mangalore, Manipal Academy of Higher Education, Manipal, India; 2 Princess Nourah Bint Abdulrahman University, Riyadh, Saudi Arabia

**Keywords:** malignancy, oral health, potentially malignant disorders, prevention, tobacco

Tobacco use continues to be one of the most significant preventable causes of morbidity and mortality on a global level, contributing to a variety of health issues, such as cancer, cardiovascular and respiratory diseases. It has a particularly severe impact on oral health, resulting in a variety of conditions that range from halitosis, stains, periodontal problems to more life-threatening issues like oral cancer. Smoking and smokeless tobacco continue to cause multifaceted damage to the oral cavity, despite the numerous regulatory and public health initiatives that have been implemented. This editorial offers a contemporary narrative that explains pathology, molecular biology, epidemiology, occupational health, and prevention strategies. The impact of tobacco on oral health is not an isolated issue, but rather a systemic, progressive, and interlinked one that must be addressed.

## Tobacco related oral mucosal lesions and malignant potential

Evidence from the population based studies revealed that chewing tobacco was significantly associated with the development of oral mucosal lesions. Descriptive cross-sectional survey on quid usage and its possible association with the occurrence of oral mucosal lesions contribute to a deeper understanding of the complex interplay between quid usage patterns and the development of oral mucosal lesions (Almalki et al.). The findings reveal significant associations between different types and forms of quid (a form of smokeless tobacco) usage and the development of lesions such as Leukoplakia, Oral submucous fibrosis, Quid-induced lichenoid reaction and Tobacco pouch keratosis. This study found a significant frequency of chewing tobacco usage, particularly among men from lower socioeconomic strata in Rajasthan, India.

These findings are consistent with those of *Investigating the association between tobacco use and oral health among security guards at a tertiary healthcare centre in New Delhi: a cross-sectional study*, which found that high-risk occupational categories like as security guards had a higher prevalence of Oral Potentially Malignant Disorders (OPMD) than non-tobacco users. The link between tobacco use and OPMDs confirms the previously documented link between tobacco and poor oral health outcomes (Chauhan et al.).

Furthermore, a large population-based screening study, *Clinical and histopathological correlation of oral malignancy and potentially malignant disorders based on a screening program at high-risk population in Tamil Nadu, India* identified subjects who underwent biopsy, Leukoplakia was the most common lesion observed among tobacco users and study stresses the need for sustained oral cancer screening as part of national health survey, thereby underscore the public health significance of the issue (Iyer et al.). These findings collectively reinforced that tobacco-related oral lesions are extremely prevalent and poses an increased risk of malignant transformation if left undiagnosed or addressed.

## Biological pathways of tobacco induced oral diseases

Chemicals included in tobacco contribute to an excessive amount of Reactive Oxygen Species (ROS), which alter the functioning of cells and impact key pathways. This results in cellular ageing, damage to DNA, and hyperinflammation, which can lead to pulmonary and oral diseases. Imdad et al. found that smokers had a higher percentage of ROS in comparison to non-smokers. The CYR61 gene was also overexpressed in smokers as compared with non-smokers. Additionally, it was found that young smokers had a considerably higher percentage of ROS and up-regulated mRNA levels of the CYR61 gene than older smokers when comparing ROS and cellular ageing. Smokers’ oral mucosal cells have been found to produce more ROS and age more quickly. This is extremely concerning and may be a substantial contributing factor to oral diseases among smokers, implying that early initiation of smoking may cause biological damage to oral tissues.

The expression of tumour necrosis factor α (TNF-α) has been demonstrated to be elevated in advanced malignancies, and it has been shown to be linked to chronic inflammation. An increase in the TNF-α concentration with increasing clinical stage suggested a role for TNF-α in the spread of OSMF involvement in anatomical structures of the oral cavity and oropharynx. Although TNF-α does not function as a valid diagnostic biomarker, the study demonstrated a positive association between TNF-α and rising stages of OSMF, which increases the biological plausibility relating tobacco, inflammatory conditions, and malignant transformation Shaikh et al.

## Tobacco induced oral microbiome dysbiosis

The microbial ecology of the mouth is disrupted by the many toxicants found in tobacco smoke that come into close association with the bacteria in the oral cavity. These harmful substances, which include N-nitrosamines and polycyclic aromatic hydrocarbons, impede DNA repair and initiate the development of tumours. Oral microbiome is an important mediator between tobacco exposure and diseases.

Senaratne et al. found that tobacco users had higher levels of bacterial richness and diversity. Fusobacteria and Actinobacteria at the phylum level and Streptococcus, Prevotella, and Veillonella at the genus level were the most prominent bacterial taxa that were enriched in users. Tobacco users had higher levels of xenobiotic biodegradation pathways and amino acid metabolism than non-users. These microbial alterations allow for xenobiotic mechanism, as well as a distinct shift toward a proinflammatory environment. Dysbiosis is not simply a secondary phenomenon, but actively involved in the initiation and progression of the disease, emphasising the need for microbiome research in tobacco-related oral health measures.

## Early prevention of second-hand exposure

Tobacco is not only detrimental to those who use it, but its smoke affects those who inhale it as well. *Effect of educational intervention in reducing exposure to second hand tobacco smoke among 12-year-old children as determined by their salivary cotinine levels and knowledge, attitude and behavior—a randomized controlled trial* revealed that all subjects included in the study had been exposed to tobacco smoke at home, which was concerning Rao et al.

Encouragingly, structured health education in schools has been proven to be beneficial in enhancing adolescents’ knowledge, attitude, and avoidance behaviour regarding second-hand smoking. This highlights the effectiveness of the educational intervention program and underscores smoke-free air is a fundamental human right, and every individual has the right to breathe clean air. Furthermore, early, preventative, and behavior-focused interventions play a vital role in preventing tobacco harm transmission between generations.

## Implications for practice and policy

Collectively, these studies highlight several significant problems. Tobacco cessation should be incorporated in routine oral health care, and dental professionals should play an active role in screening, counselling, and referral. The next step is to implement systematic, risk-based screening or health promotion programs, especially in high-burden occupational settings and schools. These are critical for early identification and prompt care of the conditions. Moreover, public health efforts need to expand beyond awareness campaigns to address this critical problem, in which social, occupational, and cultural variables play key roles.

Furthermore, clinical, genetic, and microbial studies indicate that tobacco-related oral disorders are a continuous process. The process starts with biochemical and microbiological alterations, then proceeds to clinical lesions, and finally malignant lesions. Breaking this continuous chain require a well-coordinated plan of action involving dentistry, public health policy, education, and research.

## Conclusion

The oral cavity is the initial point of interaction with tobacco and serves as an early warning system for its harmful effects. The research’ cumulative findings demonstrated that tobacco in all forms has an impact on oral and general health via structural, biological, and ecological mechanisms. Addressing this serious issue requires early detection, timely management, and strong policy. This will assist towards lowering the global morbidity and mortality caused by tobacco.

